# Application of a physiologically based pharmacokinetic model in predicting captopril disposition in children with chronic kidney disease

**DOI:** 10.1038/s41598-023-29798-0

**Published:** 2023-02-15

**Authors:** Sundus Khalid, Muhammad Fawad Rasool, Imran Masood, Imran Imran, Hamid Saeed, Tanveer Ahmad, Nawaf Shalih Alqahtani, Fahad Ali Alshammari, Faleh Alqahtani

**Affiliations:** 1 Department of Pharmacy Practice, Faculty of Pharmacy, Bahauddin Zakariya University, Multan, 60800 Pakistan; 2 Department of Pharmacy Practice, Faculty of Pharmacy, The Islamia University of Bahawalpur, Bahawalpur, 63100 Pakistan; 3 Department of Pharmacology, Faculty of Pharmacy, Bahauddin Zakariya University, Multan, 60800 Pakistan; 4 Section of Pharmaceutics, University College of Pharmacy, Allama Iqbal Campus, University of the Punjab, Lahore, 54000 Pakistan; 5 Institute for Advanced Biosciences (IAB), CNRS UMR5309, INSERM U1209, Grenoble Alpes University, 38700 La Tronche, France; 6 Department of Pharmacology and Toxicology, College of Pharmacy, King Saud University, Riyadh, 11451 Saudi Arabia

**Keywords:** Paediatrics, Therapeutics, Pharmacology, Clinical pharmacology, Pharmacokinetics

## Abstract

Over the last several decades, angiotensin-converting enzyme inhibitors (ACEIs) have been a staple in the treatment of hypertension and renovascular disorders in children. One of the ACEIs, captopril, is projected to have all the benefits of traditional vasodilators. However, conducting clinical trials for determining the pharmacokinetics (PK) of a drug is challenging, particularly in pediatrics. As a result, modeling and simulation methods have been developed to identify the safe and effective dosages of drugs. The physiologically based pharmacokinetic (PBPK) modeling is a well-established method that permits extrapolation from adult to juvenile populations. By using SIMCYP simulator, as a modeling platform, a previously developed PBPK drug-disease model of captopril was scaled to renally impaired pediatrics population for predicting captopril PK. The visual predictive checks, predicted/observed ratios (ratio_pred/obs_), and the average fold error of PK parameters were used for model evaluation. The model predictions were comparable with the reported PK data of captopril in mild and severe chronic kidney disease (CKD) patients, as the mean ratio_pred/obs_ C_max_ and AUC_0−t_ were 1.44 (95% CI 1.07 − 1.80) and 1.26 (95% CI 0.93 − 1.59), respectively. The successfully developed captopril-CKD pediatric model can be used in suggesting drug dosing in children diagnosed with different stages of CKD.

## Introduction

There is a worldwide increase in the prevalence of chronic kidney disease (CKD) as its overall annual incidence is around 8–13.5%^1–4^, and it is widespread in children. The children have a pooled renal failure (acute) incidence of 33.7%, while in the case of CKD, the data showed that 15 to 74.7 cases of pediatrics were affected with this disease per million people^5,6^. One of the most significant risk factors for accelerating the loss of kidney function is systemic arterial hypertension, which is a frequent consequence of chronic renal failure^7,8^. Reduced renal clearance as a result of a decrease in glomerular filtration rate (GFR) is a well-known effect of CKD. There are many published recommendations for drug dosage for patients with impaired renal function, but several routinely used drugs lack sufficient data to support their use in this condition^9^. When administering drugs to children, it is also important to consider how maturation affects drug disposition and action. Over time, there has been an increase in evidence demonstrating how growth and development affect drug absorption, distribution, metabolism, and excretion (ADME)^10^. Pediatric patients have distinct pathophysiologic and pharmacokinetic (PK) mechanisms than adults, which leads to a significant problem as changes in gastric pH, gastric emptying time, drug distribution, and elimination rate are different in pediatrics; as a result, it is very important to effectively determine the safe and effective drug doses to be administered in this special population^10–12^.

The kidneys are crucial for the excretion of drugs, and this is why clinicians are always focusing on maintaining renal functions in patients with different cardiovascular disorders. The angiotensin-converting enzyme inhibitors (ACEIs) are known to reduce proteinuria and albuminuria while they also slow the course of renal impairment in people with hypertension and renal failure^13–16^. All types of chronic renal failure can be delayed from reaching end-stage renal failure by using ACEIs, according to preliminary studies^17^. However, the effect of renal impairment on a drug’s PK is not confined to drug removal from the body by kidney excretion. Drug disposition in the body is determined by several distinct steps, including absorption from an extravascular site, distribution to various tissues, and elimination from the body. Over the last several decades, ACEIs have been a staple in the treatment of children as routine pharmacotherapy for newborns with several disorders^18,19^. One of the ACEIs, Captopril, is projected to have all the benefits of traditional vasodilators in the treatment of essential hypertension, congestive heart failure (CHF) as well as renovascular disorders^20–22^. Captopril-induced inhibition of ACE activity has been demonstrated to last longer in individuals with renal failure than in healthy people. This prolongation results from persistent plasma drug levels based on the delayed renal elimination of captopril in the diseased population^23,24^. In infants, plasma renin activity and angiotensin II receptor expression are all much greater than in later stages of human life, and they decline during the first years of life. As a result, newborns are more susceptible to the administration of ACEI than adults. In this age range, captopril appears to function for a longer period. Therefore, ACEI should be administered at much lower initial doses and at a slower rate in preterm and newborn infants than in adults^25–27^.

When choosing and administering drugs to children with kidney disease, clinicians should take PK changes into account to reduce toxicity and maximize pharmacological effectiveness in this population. To minimize the number of pediatric patients necessary for clinical trials and to identify safe and effective pediatric dosages, modeling, and simulation methods have been created^28^. Physiologically based pharmacokinetic (PBPK) modeling is one such technique. Based on age-related changes in patient physiology and drug pharmacokinetics, the PBPK method is a well-established method for predicting the impact of drug-drug interactions and drug exposure in adult and pediatric populations^29,30^. PBPK modeling is a mechanistic technique for investigating drug pharmacokinetics that permits extrapolation from adult to juvenile populations based on physiological variations that vary with age^31^.

There is no prior report of a PBPK model, that has been used for the prediction of captopril PK in the pediatric population. Keeping in view the importance of captopril in pediatric hypertensive patients with renal impairment, if a PBPK model is developed and evaluated in this population, it can assist clinicians in optimizing its dose in children. In order to represent age-related physiological changes in children with CKD, the study's objective was to extrapolate an existing adult PBPK model. In the present study, an already developed and evaluated PBPK drug-disease model of captopril^32^ was scaled to the pediatric population for estimating captopril ADME in children with renal impairment. The study aimed to predict captopril PK in children with renal impairment after integrating relevant pathophysiological changes.

## Methodology

### Modeling strategy

The population-based simulator SIMCYP version 21 release 1 (SIMCYP Limited, Certara, Sheffield, UK) was used to develop the pediatric PBPK model. The previously reported adult PBPK model of captopril that was successfully evaluated in adults with CKD^32^ was scaled to the pediatric population to determine the ADME of captopril in various age groups. As reported previously, the PBPK models in renally impaired children were developed by adopting the strategy of extrapolating the model from adults to pediatrics^33^. Moreover, several other studies have represented the pediatric PBPK models by implementing the same strategies used in adult PBPK models^34,35^.

**Figure 1:**
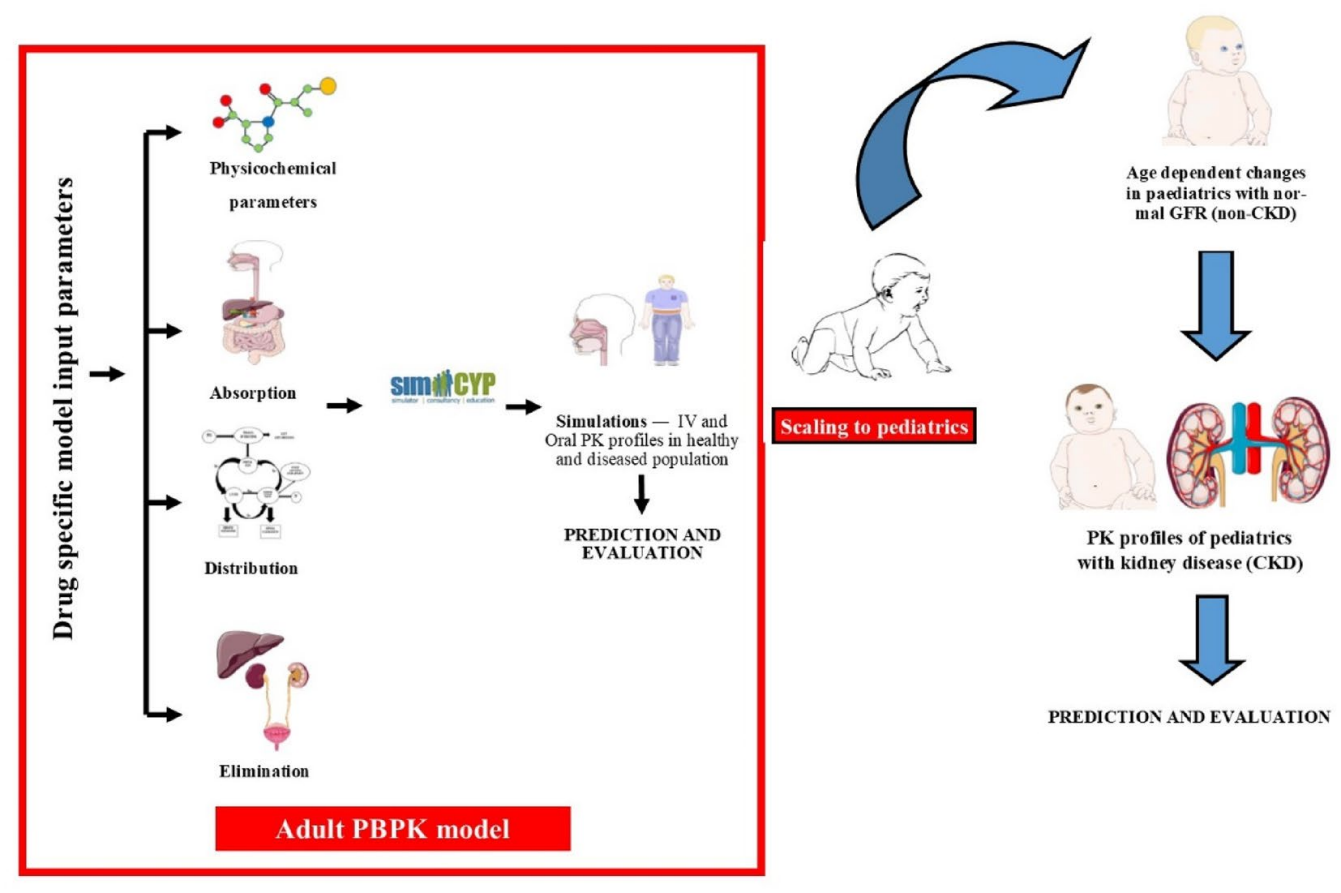
Systematic flow diagram for the captopril PBPK model developement in pediatric populations. Intervenous (IV), Pharmacokinetics (PK), Glomerular Filteration Rate (GFR), Chronis Kidney disease (CKD). Parts of the figure were drawn by using pictures from Servier Medical Art. Servier Medical Art by Servier is licensed under a Creative Commons Attribution 3.0 Unported License (https://creativecommons.org/licenses/by/3.0/).

The SIMCYP virtual pediatric population was used to simulate captopril ADME in children. This virtual population incorporates all the age-related relevant changes in demographics (height, weight, etc.), tissue composition, organ sizes, tissue flow rate, renal function, liver blood flow and gastric residence time, the abundance of metabolic enzymes, and the concentration of plasma proteins^36,37^. The following presumptions and factors were considered in the manuscript workflow while translating the adult PBPK model to pediatrics. Firstly, the contributing pathways in children and adults are qualitatively equivalent when extrapolating the adult model to pediatrics. Secondly, in the pediatric model, no additional alterations to model parameters were permitted except serum creatinine and BSA-based equation in children under 15 years of age. Lastly, the pathophysiology of CKD was assumed to be the same in children as in adults. The workflow illustration in Fig. 1 showed extrapolation of the PBPK adult model to pediatrics with normal GFR values before moving on to pediatrics with CKD by following the previously developed pediatric PBPK model-building strategy^38,39^.

### Model parameterization

In the present study, an already developed and evaluated captopril-CKD PBPK model was scaled to pediatric CKD patients by using the pediatric module of the SIMCYP simulator^32^. In the reported PBPK model, the CL_iv_ and CL_R_ values were incorporated as 49.5 L/h and 22.2 L/h, respectively. The additional elimination (27.3 L/h) was allometrically scaled in the pediatric captopril PBPK model, but no ontogeny was applied in the developed pediatric model. The model input parameters have been provided in Supplementary Table S1.

### Clinical pharmacokinetic data

An online literature search was conducted through search engines Pubmed, Google Scholar, & EBSCO for identifying and selecting the studies reporting systemic concentration versus time data and PK parameters for captopril. Moreover, age, sex, weight, and dosage forms were also taken into consideration before the selection of the studies. Finally, 02 studies were selected comprising of 14 pediatric patients, among them, 06 patients had systemic concentration profiles and the remaining 08 had clear reported values of different PK parameters (AUC, C_max_, CL). The demographic information used to evaluate the developed pediatrics model is shown in Table 1.

**Table 1:** Pediatric data used for the development of the PBPK model.

	Population	No. of patient	Dose (mg)	Age (years)	Weight (kg)	Gender	Serum creatinine (mg/dl)	eGFR^b^ (ml/min/1.73m^2^)		Refs.
1	Renal failure	1	2.3^a^	5.5	–	M	0.6	161		^40^
		1	0.8^a^	3.5	–	M	1.8	59		
		1	1.6^a^	9	–	M	3.2	20		
		1	1.6^a^	12.5	–	M	3.4	26		
		1	1.2^a^	11.5	–	M	1.3	83		
		1	2^a^	20	–	M	4.2	20		
2	Renal scarring	1	12.5	5	18	F	0.28	187		^41^
		1	20	12	29.6	F	0.56	142		
		1	16.5	6	24	F	0.35	173		
		1	23.5	10	34	F	0.67	135		
		1	46	18	66	M	1.25	83		
		1	37.5	14	52.8	F	0.58	138		
		1	37.5	11	53.7	F	0.56	148		
		1	20	7	29.7	M	0.34	200		

^a^DOSE unit in mg/kg.

^b^Estimated Glomerular Filtration rate (eGFR), Jellife equation used for GFR calculation; GFR = 98 − [(0.8)(age-20)]/ Scr (For women; GFR × 0.9).

Female (F), Male (M).

### Pathophysiological changes in renal impaired pediatrics

The pathophysiological changes occurring in CKD can affect the PK of the administered drugs. These changes have already been reported in different model-based PBPK studies^42^. The most prominent pathophysiological changes in CKD include altered drug absorption due to changes in gastric emptying time, changes in plasma protein binding, and alteration in renal blood flow which have a direct impact on renal clearance^42,43^. In pediatrics, it has been seen that the expression of renal transporters is dependent on age after birth and there is limited information on the ontogeny of the transporters responsible for tubular reabsorption in this population^44–46^. It is unclear how the transporter activity affects active tubular secretion and renal clearance in the pediatric population^47^. Since no CKD-specific disease data were available in the pediatric population, therefore the disease-related changes from the adult disease model were selected in the developed pediatric CKD model by the same underlying physiopathologic pathways as in the adult population^32^.

The kidney function in healthy population was represented by GFR > 90 ml/min/1.73 m^2^ while the damage to the kidney was classified into four major stages as mild renal failure (GFR 60 −  < 90 ml/min/1.73 m^2^), moderate renal failure (GFR 30 −  < 60 ml/min/1.73 m^2^), severe renal impairment (GFR 15 −  < 30 ml/min/1.73 m^2^) and end-stage renal disease (GFR < 15 ml/min/1.73 m^2^) ^48^. The current pediatric PBPK model represented two categories of CKD (mild and severe). By referring to the previous CKD model criteria of adults^32^, about 20% and 48–80% of non-renal CL were reduced in mild and severe renal impairment, along with the decline in GFR, respectively^49,50^. In the present study, the non-renal CL used for captopril PK prediction in mild and severe renal impairment was 80% and 60% of the value used in the healthy population. The changes in hematocrit, plasma protein binding, and gastric emptying time^42^ were also incorporated in the developed pediatric CKD model. The SIMCYP pediatrics predict GFR by BSA (body surface area) based equation in the population until 15 years of age, and above 15 years of age, GFR predictions are based on creatinine values. All the relevant disease changes of the previous adult model (i.e. changes in GFR, serum creatinine, non-renal clearance, Kp scalar) were implemented in the developed model^32^.

### Pediatric PBPK model evaluation

For each systemic concentration profile, a virtual pediatric population of 100 individuals was created by using the same demographic data as reported in the reference study (Table 1). The evaluation of the pediatrics captopril PBPK model was performed with visual predictive checks and reported PK parameters which include observed and predicted values of maximum plasma concentration (C_max_), area under the curve (AUC_0−t_), and oral clearance (CL/F). The values for these PK parameters were obtained after carrying out a non-compartmental analysis (NCA) by using the Microsoft Excel add-in program PK SOLVER^51^. Additionally, the fold-error (ratio_pred/obs_) and average fold error (AFE) for the PK parameters were also calculated for pediatric PBPK model evaluation. They were calculated separately for each population using Eqs. (1) & (2).

Mean observed/predicted ratio or Fold error:

1 $$ Fold\;Error\;/ratio_{{\left( {\frac{obs}{{pred}}} \right)}} = \frac{Predicted \;value \;of\; PK\; parameter}{{observed \;value \;of \;PK \;parameter}} $$

Average fold error:

2 $$ AFE = 10^{{\frac{{\sum \log \left( {fold\; error} \right)}}{{\text{N}}}}} $$

A two-fold error range (0.5 − twofold) was used as a reference for the evaluation of ratio_pred/obs_ of the PK parameters^32,34,52^. The previously reported PBPK models on the pediatric population have evaluated their model based on a two-fold error range criteria^53,54^. Some researchers have used a wider range (3 folds)^55^, while others have used a narrow range (1.5 fold)^56^, but the most common error range used by the researchers is a twofold error range^57–60^. The presented PBPK model predictions were evaluated by using the twofold error range. The fold error and AFE were used to assess the model accuracy while comparing the PK parameters. The fold error shows the prediction accuracy of each data point, and AFE indicates the under- and overestimation of predicted data compared to the observed values. For successful model evaluation of the pediatric PBPK model, the fold error should be in the range of 0.5–2.0 (twofold error) and on the other hand, AFE should be near the value of 1, which can be verified by previously developed PBPK models^35,52,58^.

## Results

**Figure 2:**
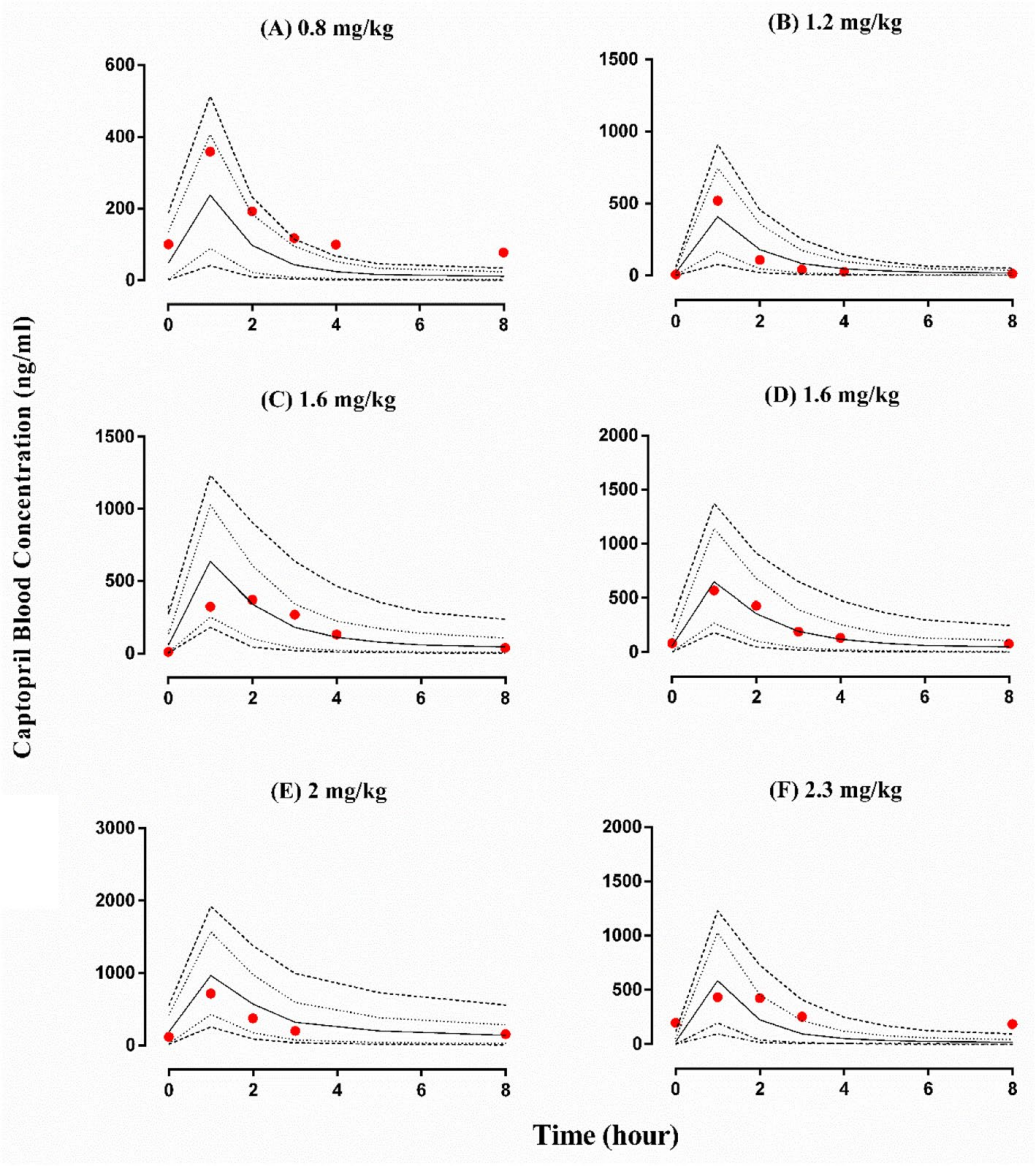
Captopril blood concentration vs time profiles in 6 pediatric patients with renal failure^40^.- (---) maximum predicted concentration, - (-·-) minimum predicted concentration, - (- -)5th percentile, - (· ··) 95th percentile, - (—) mean predicted concentration, - (•) observed values.

**Figure 3:**
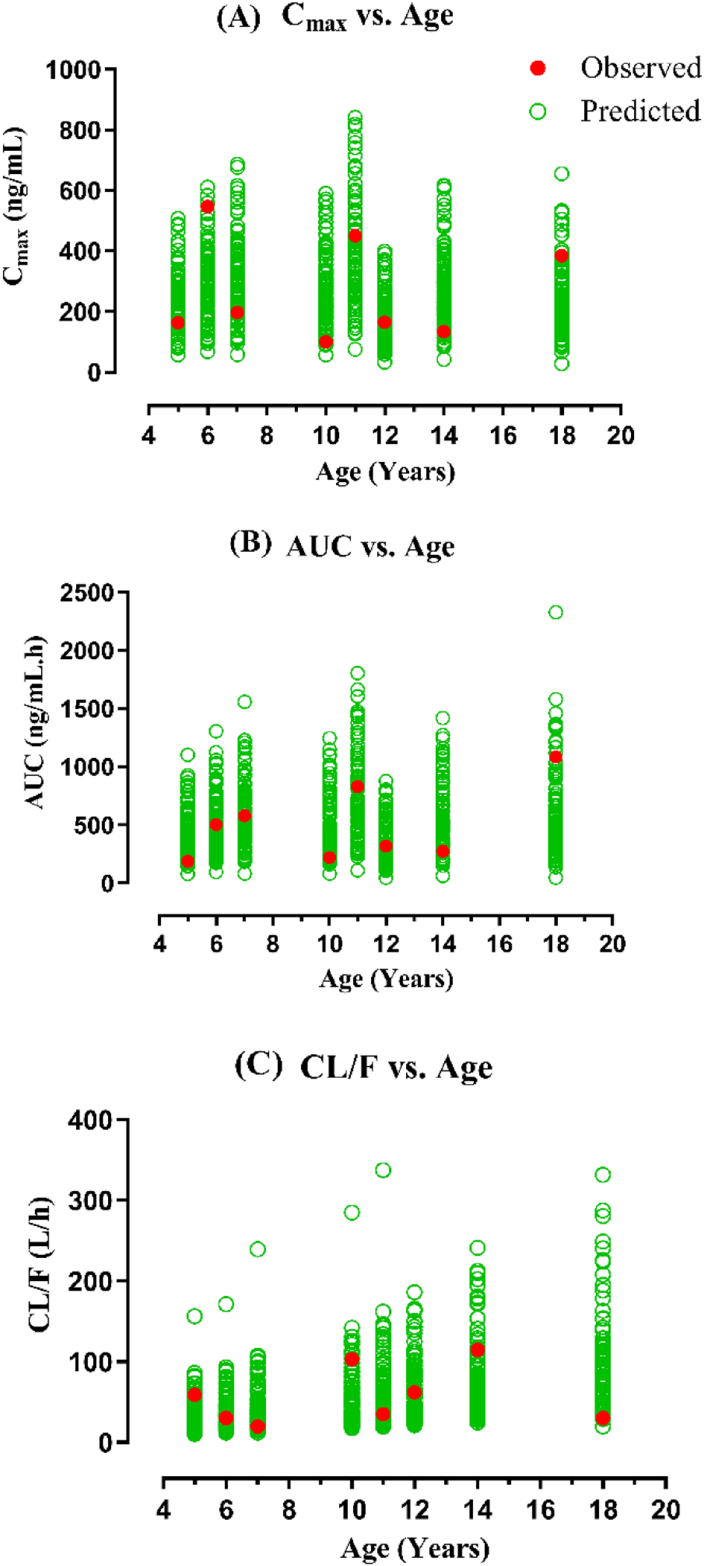
The assessment of observed and predicted PK parameters vs age of 8 pediatric patients with renal scarring. (**A**) Maximum plasma concentration (C_max_) vs age, (**B**) Area under the concentration–time curve (AUC_0−t_) vs age, (**C**) Oral Clearance (CL/F) vs age, Age range; 5‒18 years, dose range; 12.5‒46 mg.

**Figure 4:**
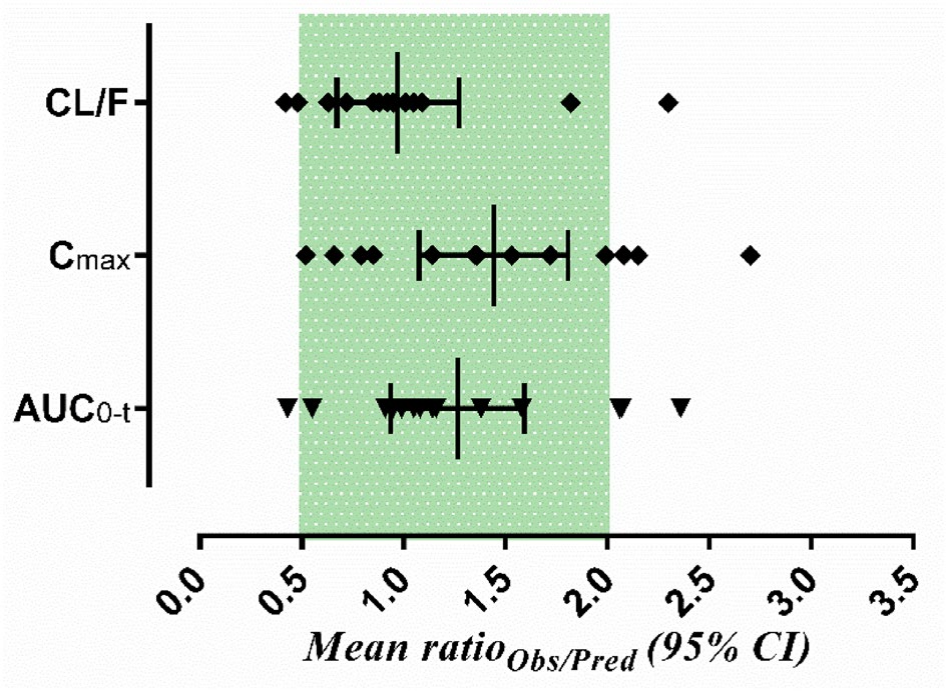
The predicted/observed ratio with their 95% confidence intervals in the 14 pediatrics with renal failure and renal scarring. AUC_0−t_; Area under the concentration–time curve, C_max_; Maximum systemic concentration, CL/F; oral clearance.

The observed and predicted blood concentration of captopril in renal impaired pediatrics and PK parameters concerning age with the dose of 2‒2.3 mg/kg and 12.5‒46 mg, respectively, are shown in Figs. 2 and 3. The visual predictive checks showed that the developed model successfully predicted captopril PK after its oral administration. The mean ratio_pred/obs_ C_max_ and AUC_0−t_ were 1.44 (95% CI 1.07 − 1.80) and 1.26 (95% CI 0.93 − 1.59), respectively. Most of the PK parameters were within the range of two-fold error except for a few values that were within 2.4 fold error (Tables 2 and 3, Fig. 4). It can be seen that the model has efficiently described captopril PK in the pediatric population, as the observed and predicted PK parameters were in agreement. Furthermore, to assess the model predictions, a comparison of the observed systemic concentrations vs. time points with the predictions was also performed. It was seen that more than 80% of the data point were within the 90th prediction interval (Supplementary Table S2).

**Table 2:** Ratio_pred/obs_ of PK parameters in the pediatric population after oral administration of captopril^40,41^.

Age	Dose (mg)	AUC_0−t_ (ng/ml × h)			C_max_ (ng/ml)			CL/F (L/h)		
		Obs	Pred	Ratio	Obs	Pred	Ratio	Obs	Pred	Ratio
*Sinaiko et al.*										
3.5	0.8^a^	1126.50	488.34	0.43	359	238.22	0.66	0.71^b^	1.64^b^	2.29
11.5	1.2^a^	768.35	832.62	1.08	520	408.69	0.79	1.56^b^	1.44^b^	0.82
9	1.6^a^	1382.10	1568.91	1.14	371	636.97	1.72	1.16^b^	1.02^b^	0.72
12.5	1.6^a^	1726.85	1637.16	0.95	569	649.95	1.14	0.93^b^	0.98^b^	0.81
20	2^a^	2153.50	2978.25	1.38	719	969.29	1.35	0.09^b^	0.07^b^	0.47
5.5	2.3^a^	2299.00	1259.76	0.55	435	585.29	1.35	1.00^b^	1.83^b^	1.82
*Levy et al.*										
5	12.5	189.40	436	2.30	163	236	1.45	66.00	28.65	0.43
6	16.5	503.50	527.44	1.05	547	283.26	0.52	32.77	31.28	0.95
12	20	318.80	361	1.13	165	182	1.10	62.74	55.36	0.88
10	23.5	221.90	523.00	2.36	100	270.00	2.70	105.90	44.93	0.42
7	20	579.80	571.64	0.99	197	301.67	1.53	34.49	34.99	1.01
14	37.5	274.50	564.93	2.06	134	278.39	2.08	136.61	66.38	0.49
11	37.5	828.40	753.17	0.91	450	384.55	0.85	45.27	49.79	1.09
18	46	1082.10	635.37	0.60	385	244.50	0.64	42.51	72.40	1.70

The area under the concentration–time curve (AUC), maximum systemic concentration (C_max_), oral Clearance (CL/F), Observed (Obs), Predicted (Pred).

^a^Dose unit in mg/kg.

^b^Clearance units in L/h/kg.

**Table 3:** Mean ratio_pred/obs_ and average fold error (AFE) values of captopril PK parameters in pediatrics population after oral administration.

Parameters	Mean ratio_pred/obs_	AFE
Oral captopril administration		
AUC_0−t_	1.26	0.84
C_max_	1.44	1.30
CL	0.98	0.87

## Discussion

In this study, a pediatric PBPK model was developed to predict the ADME after oral captopril administration in renally impaired patients. Since no clear disease-specific pathophysiological changes occurring in the pediatric population have been reported in the literature, therefore an already reported adult captopril-CKD PBPK model was extrapolated to the pediatric population^32^.

The developed captopril PBPK model showed an acceptable arrangement of observed data with predicted profiles after oral administration of captopril in mild and severe renally impaired pediatric within the age range of 3.5‒20 years. In one clinical study, the blood sampling was done at a steady state after 3 months of captopril administration^40^. The developed model has effectively projected captopril PK at a steady state after administration of 0.8‒2.3 mg/kg captopril in pediatric patients, this can be proved by the mean observed/predicted values of PK parameters (Table 2). In another study, only PK parameters (C_max_, T_max_, AUC, and CL) in 08 pediatric patients (age range; 5–18 years) after administration of 12.5–46 mg captopril were available^41^. The predicted PK parameters were compared with the reported data which was confirmed by the mean AUC_0−t_ ratio_pred/obs_ of 1.26.

The model predictions showed a rise in captopril CL/F with age, as it was 28.65 L/h at the age of 5 years and increased to 66.38 L/h at 14 years of age because of an increase in body size with age. It is evident that by normalizing the weight of the pediatrics, the CL/F values of captopril were comparable. For example, six and 11-year children had CL/F of 1.91 and 1.72 L/h/kg, respectively. This increase in captopril CL/F was primarily brought on by an increase in drug doses (from 12.5 to 46 mg)^41^. On the other hand, with an increase in disease severity, renal failure patients exhibited a decline in predicted captopril CL/F. The predicted CL/F in pediatrics with normal renal function was 1.63 L/h/kg and was decreased to 1.28 L/h/kg in mild renal impairment and it further declined to 0.83 L/h/kg in severe renal impairment patients. This reduction in captopril CL/F is associated with the pathophysiological changes incorporated within the virtual CKD pediatric population^42^.

A rise in GFR can be seen in children with age progression, starting at birth when it is quite low (20 ml/min/1.73 m^2^) and eventually reaching the adult levels (approximately 120 ml/min/1.73 m^2^) by the age of 1 year^61,62^. In the present study, all the pediatric patients included in the model evaluation were above 3.5 years of age; therefore, no significant differences were seen in the predicted captopril CL/F. Children with severe renal impairment, aged 9 and 12.5 years, had comparable CL/F of 1.02 L/h/kg and 0.98 L/h/kg after administering 1.6 mg/kg dose of captopril, respectively. However, considerable variations in captopril CL/F are anticipated within the first few weeks after delivery because the GFR is only between 30 and 40 percent of the adult value at this stage of life^62^.

The most commonly reported acceptable limit for the evaluation of the PBPK models is the twofold error range for the ratio_pred/obs_ of the PK-parameters^34,52,63^. The PBPK models developed for evaluation of drug response in disease and special populations have shown deviation from this error range^60,64^. In the presented work, some of the ratio_pred/obs_ for the PK parameters were above the twofold error range (2.36-fold), which may be acceptable while keeping in view the heterogeneous nature of the disease (CKD) and age-related physiological changes occurring in the pediatrics.

### Limitations

Since there is no clear information available in the published literature regarding the CKD-specific pathophysiological changes occurring in the pediatric population, therefore the previously reported captopril-CKD PBPK model in adults was utilized in this study. Moreover, only two pediatric studies were identified and selected after performing an extensive literature search, therefore the presented model was evaluated by using data from these pediatric studies and it can be considered as a potential limitation. In addition, due to the availability of limited published information on the age-dependent expression of renal transporters, in the developed model, it was assumed that the CL_R_ follows the same developmental trajectory as the GFR with age. Furthermore, among renal scarring patients, as mentioned in Table 1, only one patient suffered from mild renal impairment, while the remaining individuals had normal GFR values. All the pathophysiological changes of mild renal impairment were also implemented in the patient with renal scarring in the same way as incorporated in renal failure pediatrics.

## Conclusion

The developed PBPK model has efficiently captured the ADME of captopril in pediatric-CKD patients. Keeping in view the availability of very limited captopril clinical PK data, the presented PBPK model can be used in suggesting captopril doses in pediatrics with CKD.

## Supplementary Information

Supplementary Information.

## Data Availability

All the data generated during the research is either reported in the manuscript or is provided in the supplementary file.
