# Supplementary material for: Application of a physiologically based pharmacokinetic model in predicting captopril disposition in children with chronic kidney disease
Source: Sci Rep. 2023 Feb 15;13:2697. doi: 10.1038/s41598-023-29798-0 (PMC9931704; doi:10.1038/s41598-023-29798-0)
Supplement: Supplementary file 1 — Supplementary Information. [file 41598_2023_29798_MOESM1_ESM.docx]

**Application of a physiologically based pharmacokinetic model in predicting captopril disposition in children with chronic kidney disease**

Sundus Khalid^1^, Muhammad Fawad Rasool^1^, Imran Masood^2^, Imran Imran^3^, Hamid Saeed^4^, Tanveer Ahmad^5^, Nawaf Shalih Alqahtani^6^, Fahad Ali Alshammari^6^ and Faleh Alqahtani^6^*

^1^Department of Pharmacy Practice, Faculty of Pharmacy, Bahauddin Zakariya University, 60800, Multan, Pakistan; [sunduskhalid.sk@gmail.com](mailto:sunduskhalid.sk@gmail.com) (S.K.); [fawadrasool@bzu.edu.pk](mailto:fawadrasool@bzu.edu.pk) (M.F.R)

^2^Department of Pharmacy Practice, Faculty of Pharmacy, The Islamia University of Bahawalpur, 63100, Bahawalpur, Pakistan; [drimranmasood@iub.edu.pk](mailto:drimranmasood@iub.edu.pk) (I.M)

^3^Department of Pharmacology, Faculty of Pharmacy, Bahauddin Zakariya University, 60800, Multan, Pakistan; [imran.ch@bzu.edu.pk](mailto:imran.ch@bzu.edu.pk) (I.I)

^4^Section of Pharmaceutics, University College of Pharmacy, Allama Iqbal Campus, University of the Punjab, 54000, Lahore, Pakistan; [hamid.pharmacy@pu.edu.pk](mailto:hamid.pharmacy@pu.edu.pk) (H.S)

^5^Institute for Advanced Biosciences (IAB), CNRS UMR5309, INSERM U1209, Grenoble Alpes University, La Tronche, 38700, France; [tanveer.ahmad@univ-grenoble-alpes.fr](mailto:tanveer.ahmad@univ-grenoble-alpes.fr) (T.A)

^6^Department of Pharmacology and Toxicology, College of Pharmacy, King Saud University, Riyadh 11451, Saudi Arabia; [**437101259@student.ksu.edu.sa**](mailto:437101259@student.ksu.edu.sa) (N.S.A); [**437102936@student.ksu.edu.sa**](mailto:437102936@student.ksu.edu.sa) (F.A.A); [afaleh@ksu.edu.sa](mailto:afaleh@ksu.edu.sa) (F.A)

* Correspondence: [fawadrasool@bzu.edu.pk](mailto:fawadrasool@bzu.edu.pk) (M.F.R), [afaleh@ksu.edu.sa](mailto:afaleh@ksu.edu.sa) (F.A)

Table S1. Drug-specific model input parameters used for the development of captopril PBPK model (1).

| **Parameters** | **Model input values** | **Reference** |
| --- | --- | --- |
| *Physicochemical properties* | | |
| Molecular weight (g/mol) | 217.29 | ([1](#_ENREF_1)) |
| Log*P* _o:w_ | 0.34 | ([1](#_ENREF_1)) |
| pK_a_ | 4.02 | ([2](#_ENREF_2)) |
| *Absorption* | | |
| Model: First Order | | |
| k_a_ (1/h) | 1.75 | Manual optimization* |
| t_lag_ (h) | 0.2 |  |
| f_a_ (1/h) | 0.7 | ([3](#_ENREF_3)) |
| *Distribution* | | |
| Model: Minimal PBPK | | |
| K_in_ (1/h) | 0.25 | Manual optimization* |
| K_out_ (1/h) | 0.25 |  |
| B/P ratio | 1 | ([4](#_ENREF_4)) |
| *f*_u_ | 0.73 | ([3](#_ENREF_3)) |
| V_ss_  (L/kg) | 0.26 | Prediction Method 2 Rodger and Rowland method |
| *Elimination* | | |
| CL_iv_ (L/h) | 49.5 | ([5](#_ENREF_5)) |
| CL_R_ (L/h)** | 22.2 |  |

LogPo:w; octanol-water partition coefficient, ADAM; advanced dissolution, absorption, and metabolism, P_eff_; human jejunum permeability, *f*_u_; fraction of unbound drug in plasma, V_ss_; volume of distribution at steady-state, CL_iv_; intravenous clearance, CL_R_; renal clearance

* Manually adjusted afer comparing reported and predicted data

Table S2: Comparison of observed systemic concentrations vs. time points with the predictions along with their 90^th^ prediction interval.

| **Age (years)** | **Dose**  **(mg/kg)** | **Time**  **(hours)** | **Observed values** | **5^th^ percentile** | **95^th^ percentile** | **Remarks** |
| --- | --- | --- | --- | --- | --- | --- |
| 3.5 | 0.8 | 0 | 101 | 4.01 | 135.94 |  |
|  |  | 1 | 359 | 89.05 | 407.76 |  |
|  |  | 2 | 193 | 21.95 | 184.10 |  |
|  |  | 3 | 118 | 8.18 | 95.05 |  |
|  |  | 4 | 100 | 5.04 | 52.54 |  |
|  |  | 8 | 78 | 2.33 | 24.21 |  |
| 11.5 | 1.2 | 0 | 6.7 | 3.64 | 44.68 |  |
|  |  | 1 | 520 | 168.19 | 746.44 |  |
|  |  | 2 | 108 | 46.61 | 358.61 |  |
|  |  | 3 | 41 | 17.61 | 173.26 |  |
|  |  | 4 | 28 | 9.64 | 97.45 |  |
|  |  | 8 | 13 | 2.79 | 37.07 |  |
| 9 | 1.6 | 0 | 12.2 | 10.92 | 129.67 |  |
|  |  | 1 | 325 | 254.02 | 1026.80 |  |
|  |  | 2 | 371 | 101.86 | 608.79 |  |
|  |  | 3 | 270 | 38.25 | 344.14 |  |
|  |  | 4 | 132 | 22.39 | 225.74 |  |
|  |  | 8 | 40 | 8.91 | 108.54 |  |
| 12.5 | 1.6 | 0 | 84.7 | 10.98 | 125.31 |  |
|  |  | 1 | 569 | 269.57 | 1143.15 |  |
|  |  | 2 | 431 | 105.16 | 680.51 |  |
|  |  | 3 | 191 | 42.38 | 394.07 |  |
|  |  | 4 | 135 | 23.90 | 255.83 |  |
|  |  | 8 | 78 | 9.07 | 109.61 |  |
| 20 | 2 | 0 | 119 | 27.34 | 420.04 |  |
|  |  | 1 | 719 | 429.73 | 1571.59 |  |
|  |  | 2 | 377 | 177.77 | 980.30 |  |
|  |  | 3 | 201 | 75.08 | 594.45 |  |
|  |  | 8 | 158 | 25.34 | 284.21 |  |
| 5.5 | 2.3 | 0 | 197 | 3.58 | 53.42 |  |
|  |  | 1 | 435 | 194.45 | 1029.54 |  |
|  |  | 2 | 425 | 38.36 | 453.93 |  |
|  |  | 4 | 252 | 13.44 | 217.90 |  |
|  |  | 8 | 186 | 2.79 | 43.69 |  |

Indicate the observed values are within the 5^th^ and 95^th^ percentile range

Indicate the observed values are not within the 5^th^ and 95^th^ percentile range

**References**

1. Benet LZ, Broccatelli F, Oprea TI. BDDCS applied to over 900 drugs. The AAPS journal. 2011;13(4):519-47.

2. Takubo H, Taniguchi T, Iwanaga K, Nomura Y. Evaluation of the changes in exposure to thiol compounds in chronic kidney disease patients using the PBPK model. Xenobiotica. 2021;51(1):31-9.

3. Kubo SH, Cody RJ. Clinical pharmacokinetics of the angiotensin converting enzyme inhibitors. Clinical pharmacokinetics. 1985;10(5):377-91.

4. Poulin P, Theil FP. Prediction of pharmacokinetics prior to in vivo studies. 1. Mechanism‐based prediction of volume of distribution. Journal of pharmaceutical sciences. 2002;91(1):129-56.

5. Creasey W, Morrison R, Singhvi S, Willard D. Pharmacokinetics of intravenous captopril in healthy men. European journal of clinical pharmacology. 1988;35(4):367-70.
